# Impure Chloroform

**Published:** 1882-04

**Authors:** 


### IMPURE CHLOROFORM.

At a recent meeting of the Société de Chirurgie, M. Lucas Championnière presented an interesting paper upon the subject of impure chloroform. Having observed for a long period of time the behavior of various patients while under chloroform, and the diversity of symptoms presented by each, particularly as to the time at which reaction was established, and the degree of danger attending the anesthetic state thus induced, he was led to make the chloroforming of patients a matter of careful study.

M. Championnière arrives at the conclusion that while these differences in the symptoms seen in various patients may in some cases be accounted for by individual susceptibility, they are in a great degree attributable to differences in the composition of the specimens of chloroform employed.

Chloroform as furnished to hospitals, and as obtained ordinarily from the druggist, is often far from good. He requested the surgeons to observe how many cases to whom they administered chloroform as ordinarily obtained terminated fatally, and to note if a series of accidents did not sometimes follow upon the administration of a new specimen of the drug, even when submitted to careful analysis and declared pure, when the old obtained from the same source had proved harmless.

To satisfy himself that the chloroform is often at fault, M. Championnière had placed various specimens of chloroform obtained from the hospitals and city druggists in the hands of M. Yvon for analysis. This chemist found that while most of the specimens disclosed no impurities under the usual tests, they nevertheless showed a variable point of ebullition. They were then treated with permanganate of potassium and redistilled, when they presented all the characters of a chemically pure article.

M. Championnière has from time to time freely administered the chloroform thus purified to his patients, and finds that it produces anesthesia rapidly ; that the period of insensibility is attended by no disagreeable or alarming symptoms, and that the patient awakes from it almost as suddenly and quite as pleasantly as from a natural sleep.—*Louisville Med. News.*

